# Circulating DNA as prognostic biomarker in patients with advanced hepatocellular carcinoma: a translational exploratory study from the SORAMIC trial

**DOI:** 10.1186/s12967-019-2079-9

**Published:** 2019-10-01

**Authors:** Marianna Alunni-Fabbroni, Kerstin Rönsch, Thomas Huber, Clemens C. Cyran, Max Seidensticker, Julia Mayerle, Maciej Pech, Bristi Basu, Chris Verslype, Julia Benckert, Peter Malfertheiner, Jens Ricke

**Affiliations:** 1 Department of Radiology, University Hospital, LMU Munich, Marchioninistrasse 15, Munich, Germany; 2 Eurofins Genomics Europe Sequencing GmbH, Constance, Germany; 3 Present Address: Institute of Clinical Radiology and Nuclear Medicine, University Medical Center Mannheim, Heidelberg University, Mannheim, Germany; 4 Department of Medicine II, University Hospital, LMU Munich, Munich, Germany; 5 University Clinic for Radiology, University of Magdeburg, Magdeburg, Germany; 6 Department of Oncology, University of Cambridge, Cambridge, UK; 7 UZ Leuven, Campus Gasthuisberg, Louvain, Belgium; 8 Department of Hepatology and Gastroenterology, Charité University Hospital, Berlin, Germany

**Keywords:** Liquid biopsy, Circulating tumor DNA, Hepatocellular carcinoma, Biomarkers

## Abstract

**Background:**

Liquid biopsy based on cell-free DNA circulating in plasma has shown solid results as a non-invasive biomarker. In the present study we evaluated the utility of circulating free DNA (cfDNA) and the sub-type tumor DNA (ctDNA) in hepatocellular cancer (HCC) patients to assess therapy response and clinical outcome.

**Methods:**

A cohort of 13 patients recruited in the context of the SORAMIC trial with unresectable, advanced HCC and different etiological and clinicopathological characteristics was included in this exploratory study. Plasma samples were collected between liver micro-intervention and beginning of sorafenib-based systemic therapy and then in correspondence of three additional follow-ups. DNA was isolated from plasma and next generation sequencing (NGS) was performed on a panel of 597 selected cancer-relevant genes.

**Results:**

cfDNA levels showed a significant correlation with the presence of metastases and survival. In addition cfDNA kinetic over time revealed a trend with the clinical history of the patients, supporting its use as a biomarker to monitor therapy. NGS-based analysis on ctDNA identified 28 variants, detectable in different combinations at the different time points. Among the variants, HNF1A, BAX and CYP2B6 genes showed the highest mutation frequency and a significant association with the patients’ clinicopathological characteristics, suggesting a possible role as driver genes in this specific clinical setting.

**Conclusions:**

Taken together, the results support the prognostic value of cfDNA/ctDNA in advanced HCC patients with the potential to predict therapy response. These findings support the clinical utility of liquid biopsy in advanced HCC improving individualized therapy and possible earlier identification of treatment responders.

## Background

Hepatocellular carcinoma (HCC) is the sixth most common cancer and the second leading cause of cancer-related death globally, with a median survival of only 7 months, if untreated [1–3]. Although cirrhosis, generally resulting from chronic inflammation and oxidative stress [4], is recognized as the most frequent risk factor, patients are often diagnosed with HCC in an already advanced phase, when treatment is limited and resection or transplantation are not any longer an option. This is in part due to the limited sensitivity and specificity of the standard diagnostic modalities of imaging and biomarkers, such as serum alpha-fetoprotein (AFP) [5], in the detection of small tumors during surveillance of high risk populations. Advanced HCC often shows a poor outcome, with very limited benefit from cytotoxic agents. Targeted therapy with the multi-tyrosine kinase inhibitor sorafenib has been the mainstay of systemic treatment, nevertheless resulting in modest improvements in overall survival over placebo and often only in subgroups of patients [6]. Identification of early predictors of therapy response would be highly desirable in order to move quickly patients to more effective treatments.

Circulating cell-free DNA (cfDNA) is detectable in the plasma and serum of healthy individuals as consequence of cellular necrosis or apoptosis. Cancer patients show a higher amount of cfDNA since tumor cells divide faster than normal cells, and cfDNA is released in higher proportion [7–9]. The fraction of cfDNA derived from the tumor is indicated as circulating tumor DNA (ctDNA) [10, 11]. In the last years, analysis of both cfDNA and ctDNA has gained considerable attention as novel blood biomarkers, since quantification and kinetic analysis of cfDNA [12, 13] and molecular profiling of ctDNA [14] have demonstrated both a predictive and prognostic value. Venipuncture, with capacity for serial sampling over time, may offer advantages over standard biopsy, avoiding complications from incisions and reflecting the genetic heterogeneity of the whole tumor. In addition, due to the short half-life of circulating DNA [15], genetic analysis may offer “real time” insights into the kinetic mutations arising during therapy. The prognostic value of cfDNA/ctDNA in early HCC has been previously shown [16, 17]. The aim of the present study was to evaluate if cfDNA quantification over time may be a valid monitoring strategy to predict clinical outcome in advanced HCC patients in the context of the SORAMIC trial [18, 19], and whether molecular profiling of ctDNA may track the genetic evolution of the tumor during the combined systemic and liver-directed micro-interventional treatment.

## Methods

### Patients and therapy

**Fig. 1:**
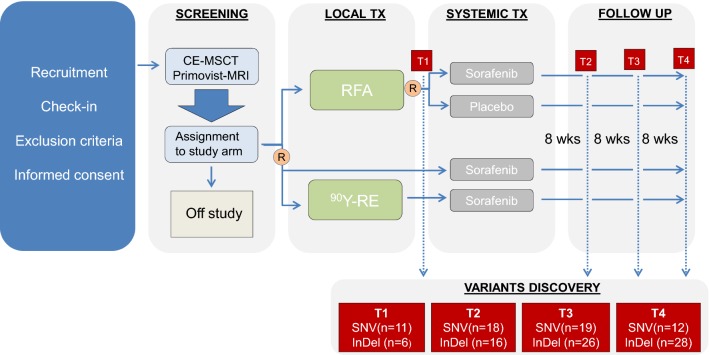
SORAMIC trial design. Indicated in red are the time points chosen for discovery of the variants. SNV, single nucleotide variant; InDel, insertion and deletion; R, randomization; TX, therapy; RFA, radiofrequency ablation; ^90^Y-RE, ^90^Y-radioembolization

A total of 13 male patients (median age (years ± SD): 68 ± 8.91, range 46–82) from the prospective randomized multicenter phase II SORAMIC trial (EudraCT 2009-012576-27, NCT01126645) were included in this exploratory study [18, 19]. Participants were recruited between 2011 and 2016 at different European study centers. Advanced, unresectable HCC patients received radioembolization with Yttrium-90 (90Y-RE) in combination with systemic treatment with sorafenib (n = 10) or radiofrequency ablation in combination with sorafenib (n = 2) or placebo (n = 1). Treatment efficacy was evaluated based on imaging and alfa-fetoprotein (AFP) [20, 21]. The median overall survival was 23.8 months (range 11.9 – 41.4) and only two patients showed recurrence with a progression free survival of 22.3 and 26.8 months. Blood samples were collected at four different time points: after micro-interventional therapy and before starting the systemic therapy with sorafenib (T1), approximately 8 weeks after beginning of systemic therapy (T2, median numbers of days between T1 and T2: 112), at first follow up (FU) (T3, median numbers of days between T1 and T3: 224) and at second FU (T4, median numbers of days between T1 and T4: 297). The SORAMIC trial design is given in Fig. 1, where the time points relative to blood collection are indicated. The study was approved by all the involved ethical boards and conducted in accordance with the Declaration of Helsinki [22]. Before entering the study, all patients gave their written informed consent.

### DNA isolation, library preparation and next-generation sequencing (NGS)

Peripheral blood (5 ml) was drawn in EDTA tubes (Becton–Dickinson, Heidelberg, Germany) and processed immediately (centrifugation 3000 rpm, 5 min, 4 °C) to collect plasma and buffy coats, which were aliquoted and stored at − 80 °C until further use. Plasma was used for extraction of cell-free DNA (cfDNA) and buffy coats were used for extraction of genomic DNA (gDNA). DNA extraction, quality control and NGS were performed at Eurofins Genomics GmbH (Konstanz, Germany). The quality and quantity of the extracted DNA was assessed via electropherograms and fluorometer concentration determinations (for details, see Additional file 1: Figure S1). The extracted DNA was used for library preparation and subsequent sequencing. A summary of the depth of coverage is shown in Additional file 2: Table S1.

The genes were selected according to their importance and in accordance with clinical guidelines such as the European Society for Medical Oncology (ESMO), the National Comprehensive Cancer Network (NCCN), and the College of American Pathologists (CAP).

**Fig. 2:**
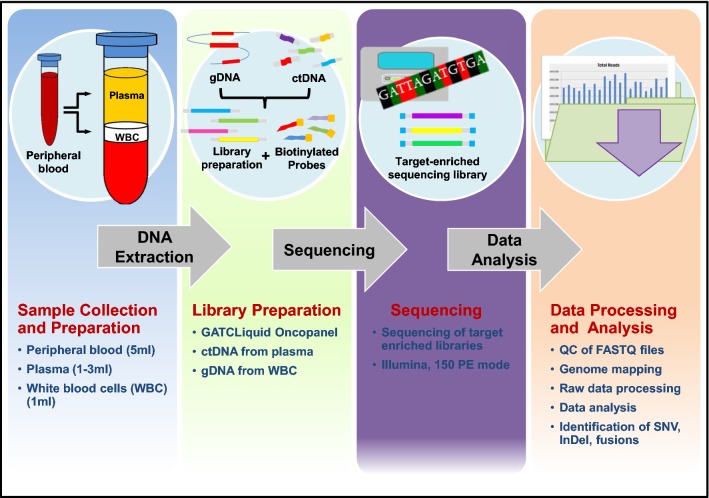
Schematic description of the experimental workflow. Peripheral blood was collected and processed to separate plasma from buffy coat containing white blood cells (WBC). Whole circulating DNA was extracted and used for library preparation. Next generation sequencing was performed on Illumina platform using the 150 paired-end (PE) mode. To distinguish somatic from germline mutations, genomic DNA extracted from WBC was analyzed in parallel

NGS was performed on Illumina platform using the 150 paired-end mode. The sequencing reads were quality checked and mapped to the human genome using BWA with default parameters [23]. After target processing and alignment refinement, variants (single nucleotide variants, SNV; Insertions and deletions, InDel; and gene fusions according to Chimer DB 2.0 [24]) were discovered and annotated. To distinguish somatic from germline mutations, genomic DNA extracted from buffy coats was analyzed in parallel (Additional file 3: Table S2). The whole experimental workflow is schematically described in Fig. 2.

### Statistical analysis

All statistical analyses were performed using IBM SPSS Statistics 21.0.0 (IBM Corporation, New York, N.Y.). Results for numerical data are given as median together with minimum and maximum of the sample (i.e. range). The correlation between tumor size and cfDNA concentrations at different time points were analyzed using the Pearson’s correlation coefficient and corresponding significance test. cfDNA at different time points were compared between patient groups featuring different clinicopathological characteristics using the non-parametric Mann–Whitney U test. The association between variants at different time points and patients’ clinical characteristics was evaluated using the Fisher’s exact test. Overall survival (OS) was analyzed using the Kaplan–Meier method, and survival estimates in different groups were compared using the log-rank test. Patients were clustered in two groups corresponding to high and low cfDNA concentration (high cfDNA = over the median, low cfDNA = below the median). OS was calculated from the date of primary tumor diagnosis to the date of death or the date of the last clinical follow-up. All tests were carried out two-sided. Due to the low sample size of n = 13 patients, no alpha adjustment was made. All statistical tests are interpreted at a significance level of alpha = 5% and according results are considered exploratory.

## Results

### Clinicopathological characteristics of the patients

Within the cohort chosen for this study, patients were divided in two subgroups: patients with an age below 65, who did not present with liver cirrhosis at time of recruitment, did not report any past alcohol abuse and, based on the SORAMIC trial [19] results, responded better to therapies; and patients over 65, who presented with liver cirrhosis at time of recruitment, reported past alcohol abuse and responded worse to therapies. None of the patients were positive for Hepatitis B, only 1 was positive for Hepatitis C, and 4 presented non-alcoholic steatohepatitis (NASH). Three patients (33.3%, for n = 4 the number of lesions was not available) presented with diffuse disease (> 20 lesions), while among the others the median number of lesions was 2 (range 0–28) and the median size of the lesions was 49 mm (range 8–160 mm). Three patients (23.0%) showed portal vein infiltration, all of them presented with a Child–Pugh A liver function, 46.1% (n = 6) of the patients were Barcelona Clinic Liver Cancer Staging (BCLC) C, while the remaining were A (n = 3, 23.0%) or B (n = 4, 30.7%). Four patients (30.7%) presented lymph node metastases and one of them (7.7%) also bone metastases. Two patients (66.6%) showed recurrence at 2 years FU. A summary of the clinicopathological characteristics of the patients is given in Table 1.

**Table 1:** Clinicopathological characteristics of the patients

ID	Age	Liver cirrhosis	HBV	HCV	Alcohol abuse	Other	Number of lesions (> 20 = DD)	Max. diameter largest lesion (mm)	PVI	CP	CP points	BCLC stage	Therapy	M	R	death	OS (months)
A	46	No	Neg	Neg	No	Nash	28	34	No	A	5	C	RE SRFB	No	No	0	40.7
B	60	No	Neg	Neg	No	–	99	160	No	A	6	C	RE SRFB	Yes	No	1	38.6
C	61	No	Neg	Neg	No	Nash	99	8	No	A	5	B	RE SRFB	No	No	0	23.4
D	61	No	Neg	Neg	No	Nash	na	88	No	A	5	B	RE SRFB	No	No	0	16.1
E	59	No	Neg	pos	No	–	na	55	Yes	A	5	C	RE SRFB	Yes	No	1	11.7
F	68	Yes	Neg	Neg	Yes	Nash	3	91	Yes	A	6	C	RE SRFB	Yes	No	1	26.8
G	75	Yes	Neg	Neg	Yes	–	na	na	No	A	6	B	RE SRFB	No	No	1	13.7
H	68	Yes	Neg	Neg	Yes	–	3	15	Yes	A	5	C	RE SRFB	No	No	1	16.8
I	66	Yes	Neg	Neg	Yes	–	na	65	No	A	5	C	RE SRFB	Yes	No	1	13.0
J	69	Yes	Neg	Neg	Yes	–	2	75	No	A	5	B	RE SRFB	No	No	1	17.5
K	70	Yes	Neg	Neg	Yes	–	1	43	No	A	5	A	RFA PLC	No	Yes	1	27.1
L	82	Yes	Neg	Neg	Yes	–	1	40	No	A	6	A	RFA SRFB	No	No	1	26.3
M	73	Yes	Neg	Neg	Yes	–	1	33	No	A	5	A	RFA SRFB	No	Yes	1	32.5

HBV, hepatitis B virus; HCV, hepatitis C virus; DD, diffuse disease; Nash, non-alcoholic steatohepatitis; PVI, portal vein infiltration; CP, Child–Pugh, BCLC, Barcelona Clinic Liver Cancer; RE, ^90^Y-Radioembolization; SRFB, sorafenib; PLCB, placebo; M, metastases; R, recurrence; OS, overall survival, in months; na, not available

### Correlation between cfDNA levels, patient’s clinicopathological characteristics and clinical outcome

**Fig. 3:**
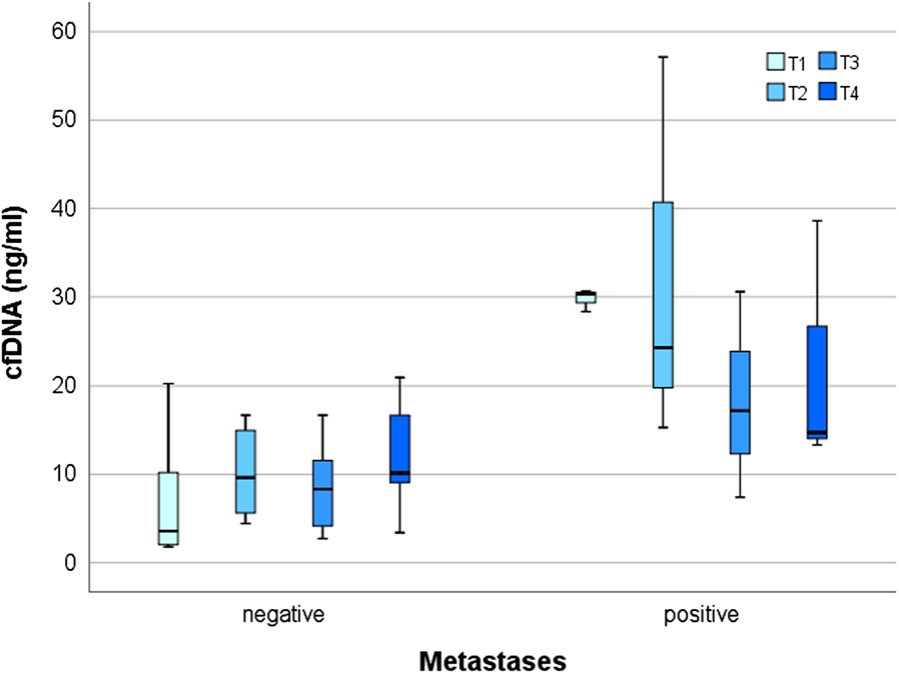
cfDNA concentrations at different time points and presence of metastases. The analysis shows that patients presenting metastases when recruited in the trial, had still a significant higher amount of plasmatic cfDNA in the timeframe between micro-interventional therapy and the beginning of sorafenib-based systemic therapy (T1, *p *= 0.012). Borderline significance (*p *= 0.073) was found after the beginning of systemic therapy (T2), while no significant difference was found at the two later time points. Comparison was performed using the Mann–Whitney U test (* ≤ 0.05)

**Fig. 4:**
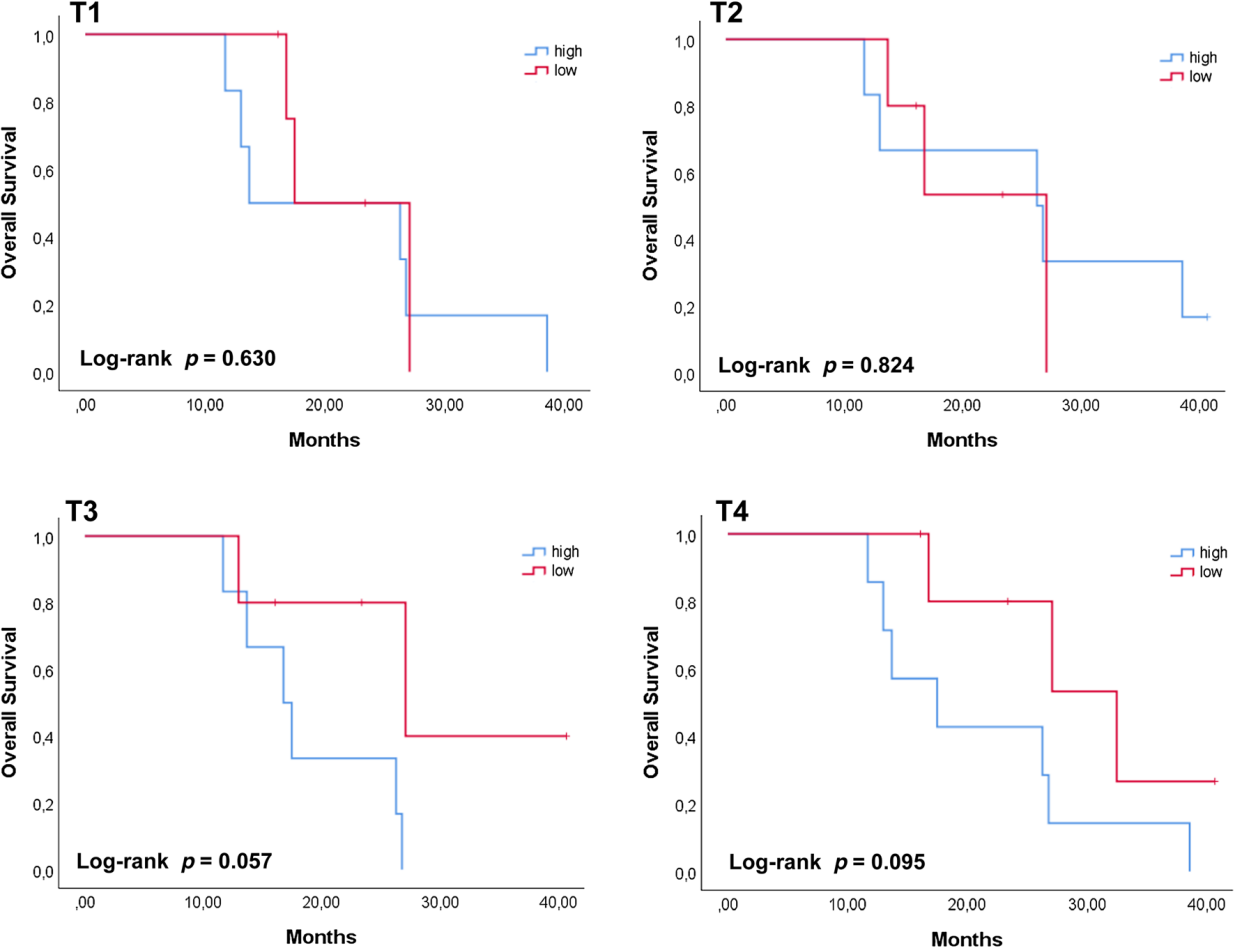
Survival plots for HCC patients grouped according to the amount of cfDNA at the different time points. Patients’ total population was grouped according to the corresponding cfDNA concentration with respect to the median value found at each time point. Higher cfDNA concentration at later time points (T3 and T4) was showing a trend with a shorter OS (*p *= 0.057 and *p *= 0.095) while no association (*p* > 0.1) was found and earlier time points (T1 and T2). High and low cfDNA levels were defined as being above or below the median values at the different time points. OS was analyzed using the Kaplan–Meier method and survival estimates in the different groups were compared using the log-rank test

cfDNA was isolated from plasma collected at the 4 time points (T1, T2, T3, and T4) during the SORAMIC trial. Nine of the 52 cfDNA samples (17.3%) were excluded from the subsequent analysis due to high genomic DNA contamination or because the concentration was below the threshold (Additional file 1: Figure S1). In the remaining samples, the amount of cfDNA at the different time points was ranging between 2.04 ng/ml to 160.75 ng/ml with a median of 10.2 ng/ml at T1, 15.3 ng/ml at T2, 9.3 ng/ml at T3 and 13.3 ng/ml at T4 (Table 2). We did not observe a significant correlation between cfDNA concentration at T1 and tumor volume or between cfDNA concentrations at any time points and patient’s clinical characteristics (all *p *>* 0.1*) (data not shown). On the contrary, we did observe a difference between the concentrations of cfDNA in the presence or absence of metastases, which was significant (*p *= 0.012) or borderline significant (*p *= 0.073) at T1 and T2, respectively (Fig. 3). The relative amount of cfDNA at the different time points was also evaluated with respect to overall survival (OS). Patients were grouped according to high or low cfDNA levels with respect to the median values at the different time points. We found a borderline significance at the latest time points (T3, *p *= 0.057; T4, *p *= 0.095), suggesting that patients keeping high levels of plasmatic cfDNA while receiving systemic therapy might have a worse outcome (Fig. 4). On the contrary we did not find any association between cfDNA levels at earlier time points and OS (all *p *> 0.1). The fact the we found a borderline significance might be due to the low number of patients included in the analysis, therefore it will be necessary to validate these preliminary results with a bigger cohort of patients.

**Table 2:** Amount of cfDNA (ng/ml) and AFP (ng/ml) in plasma samples at the different time points

Patients	T1	T2	T3	T4	Median [cfDNA]
	cfDNA	cfDNA	cfDNA	cfDNA	
	AFP	AFP	AFP	AFP	
A	na	39.44	8.39	10.11	10.1
	85.00	11.00	13.00	6.00	
B	12.78	17.68	na	15.30	15.3
	373.00	75.00	33.00	45.00	
C	4.35	7.34	2.72	3.40	3.9
	2.00	3.00	3.00	3.00	
D	2.81	14.96	4.18	9.07	6.6
	4.00	3.00	9.00	16.00	
E	30.63	15.30	17.20	38.62	23.9
	3.00	3.00	10.00	42.10	
F	28.33	57.12	30.60	14.73	29.5
	2.00	4.00	na	4.00	
G	10.20	11.90	11.56	20.94	11.7
	4.00	6.00	8.00	4.00	
H	1.81	5.64	9.38	11.02	7.5
	13.00	19.00	30.00	72.00	
I	30.37	24.28	7.41	13.33	18.8
	1681.00	1685.00	991.00	967.00	
J	7.96	na	12.60	160.75	12.6
	6154.00	2410.00	8732.00	3855.00	
K	2.04	4.42	7.28	9.25	5.8
	4.00	3.00	3.00	3.00	
L	20.26	16.68	16.68	16.68	16.8
	7.00	5.00	6.00	7.00	
M	na	na	na	8.98	8.98
	9.00	4.00	4.00	6.00	
Median [cfDNA]	10.2	15.3	9.3	13.3	

cfDNA median values are given for each patient at the four time points (last column) or for each time point for all patients (last row)

na, not available

### cfDNA in comparison to the conventional biomarker AFP

**Fig. 5:**
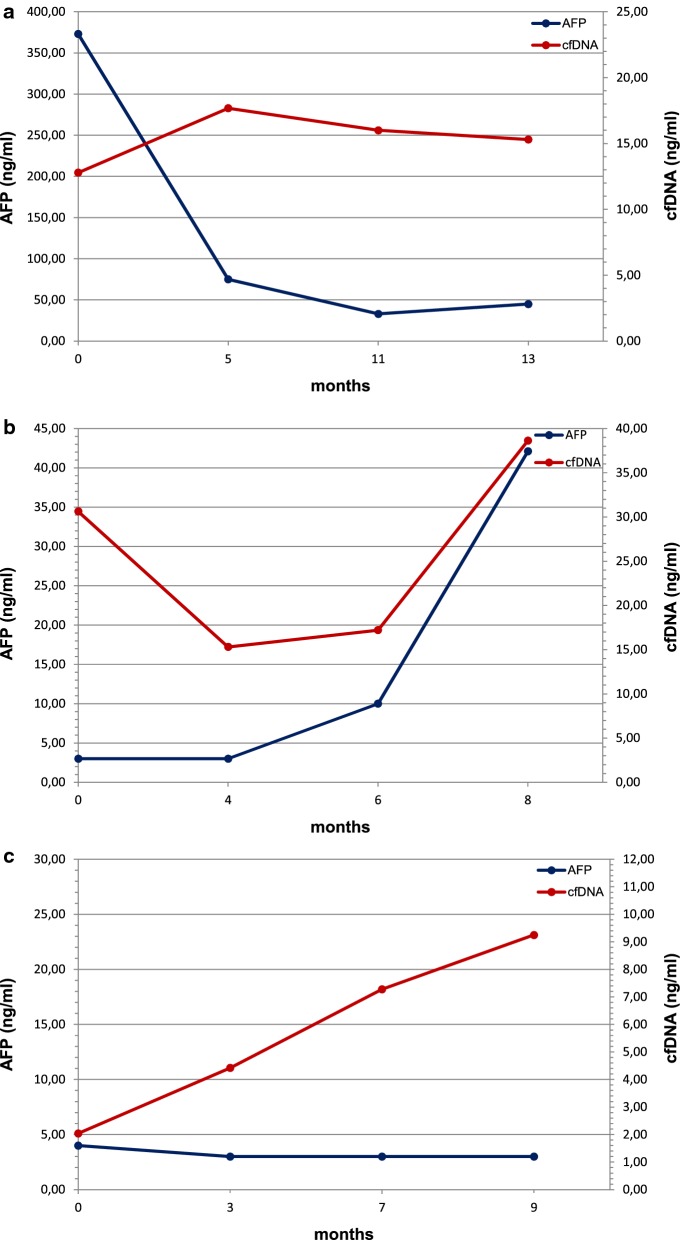
Comparison between cfDNA and AFP levels in three cases corresponding to patients showing different clinical characteristics. Panels **a**, **b** and **c** represent the described clinical cases of patients B, E and K, respectively

AFP is detectable in as many as 30% of HCC patients at time of diagnosis and usually remains low during the course of the disease, even with advanced HCC [20]. AFP > 400–500 ng/ml supports diagnosis of HCC [21]. At the considered time points, only 2 (15.4%) of the patients showed high AFP values (> 400 ng/ml), 4 (30.8%) showed values between 20 and 400 ng/ml and 7 (53.8%) showed values between 2 and 20 ng/ml (Table 2). It has been already shown that cfDNA has clinical diagnostic relevance, reflecting therapy response [25]. We report here in more detail three vignettes of patients with evidence of disease progression, for which cfDNA offered earlier monitoring value than conventional biomarkers such as AFP (Fig. 5). A concentration of 400 ng/ml was chosen as cut-off value. The first patient (patient B) entered in the trial with a diffuse disease in the liver and lymph nodes and bone metastases; he died after 38.6 months from enrolment in the SORAMIC trial. AFP concentration was high (373 ng/ml) before starting sorafenib treatment; however it decreased very rapidly below 50 ng/ml and remained low while assuming sorafenib, suggesting efficacy of treatment. On the contrary, we did not find a corresponding reduction in the cfDNA concentration which instead increased during time, suggesting the presence of progressive disease, as confirmed by CT imaging at follow up (Fig. 5a). The second patient (patient E) entered in the study with lymph node metastases, portal vein infiltration and disease progression on treatment with an OS of 11.7 months from the beginning of therapy. At time points considered, AFP values were measured always below 50 ng/ml; in contrast, cfDNA level, while showing a drastic decrease immediately after the beginning of the therapy, increased rapidly between 6 and 8 months from the beginning of the therapy, reflecting the rapid progression of the disease and indicating a change in the response to sorafenib (Fig. 5b). The third patient (patient K) entered in the study presenting only one hepatic lesion and no evidence of metastases. AFP was always measured below 5 ng/ml at all time points, however cfDNA showed a steadily increase, predicting the progression of the disease. The patient finally relapsed and died shortly after (Fig. 5c). In conclusion, our data, in line with other reports, showed improved diagnostic sensitivity of cfDNA over AFP [24, 26], suggesting also in advanced HCC cfDNA as a biomarker for monitoring therapy efficacy.

### Somatic mutation analysis

**Fig. 6:**
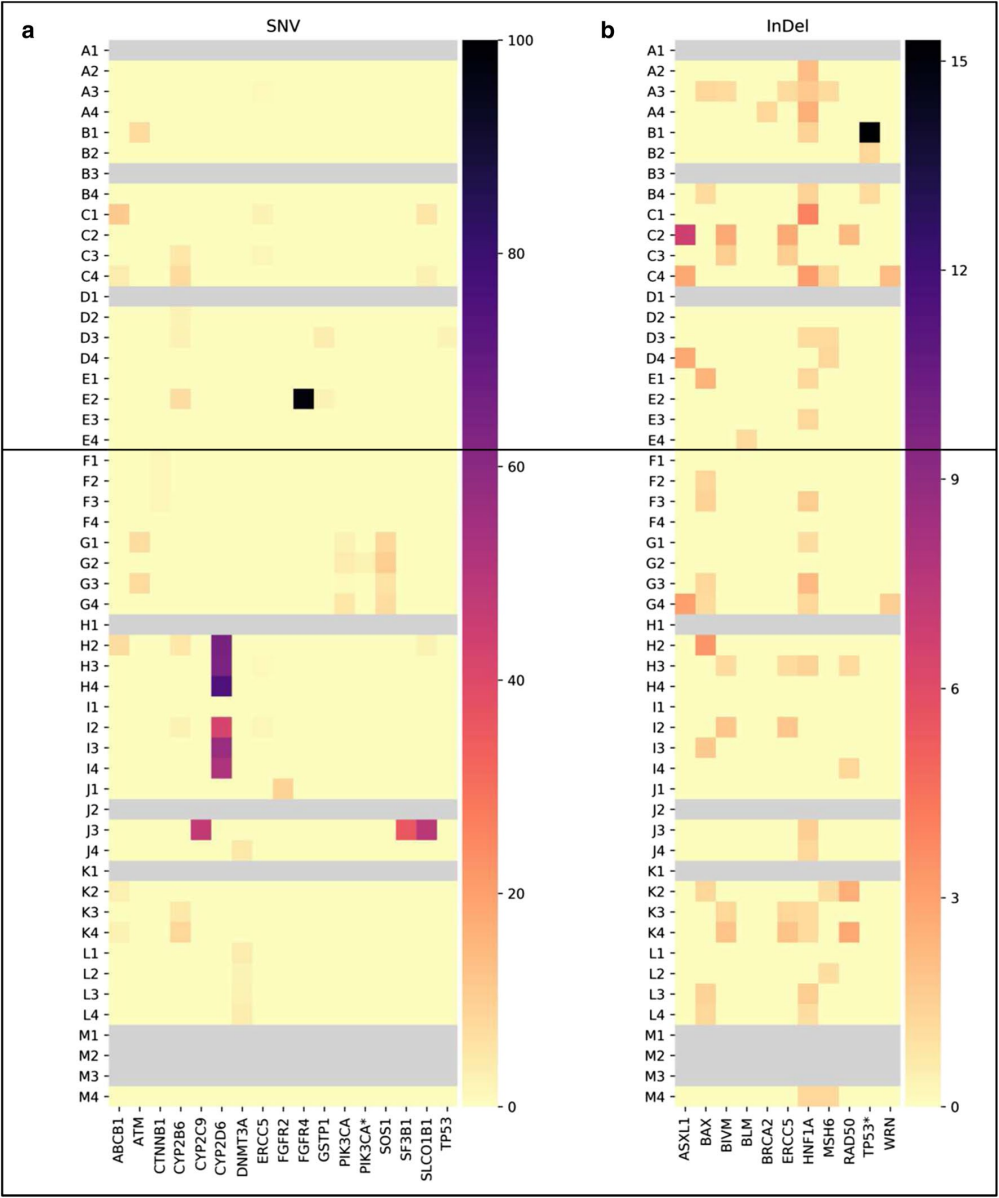
Heatmaps showing the variants discovered in the two subgroups of patients (A–E: below 65, no liver cirrhosis, no past alcohol abuse and better response to therapies; F–M: over 65, with liver cirrhosis, past alcohol abuse and worse response to therapies) at the different time points. In (**a**) and in (**b**) SNV and InDel with relative mutation frequencies are reported, respectively

Genomic profiling of ctDNA was undertaken at the different time points, with the screening of 597 cancer-relevant genes. Only those mutations proven to have clinical impact according to the ClinVar database (National Center for Biotechnology Information-NCBI) were further analyzed. In total 28 variants, including single nucleotide variations (SNV) or insertions and deletions (InDel) were discovered in our dataset, while no gene fusion was identified (Fig. 6). All patients showed at least one variant (median = 6 variants per patient, range 3–16), however patterns varied between patients. Variants were found in genes involved in DNA repair, multidrug resistance, cell cycle control, signal transduction, transcription control, chromatin structure, apoptosis and DNA methylation (Table 3). We detected somatic mutations in recognized HCC driver genes such as CTNNB1 and TP53, however at very low frequency. Over the 28 variants, three genes were showing the highest mutation allele frequency (MAF): HNF1A (n = 12, 92.3%), BAX (n = 9, 69.2%) and CYB2B6 (n = 6, 46.1%). We observed that in a high percentage of genes (n = 19, 68%) variants were found mainly after the beginning of sorafenib treatment, suggesting a possible clonal selection induced by the therapy itself. Nevertheless, a different kinetic was also observed. For example, in the case of HNF1A, 50% of the patients showed the variant already before starting to assume sorafenib. At T2, only one patient was still positive, suggesting a good therapy response. However at T3 the variant was showing a MAF of 82% (n = 9), a finding which could suggest a clonal expansion of cells not responsive any longer to sorafenib.

**Table 3:** List of variants grouped according to gene function

Gene group	Location	AA	Codon	Patients (%)
DNA repair				
ATM	chr11:108121426	–	c.1236-2A>T	2 (15.4)
BLM	chr15:91304138	p.N515fs na	c.1544delA c.*468delA	1 (7.7)
BRCA2	chr13:32954022	p.T18fs p.T3033fs	c.52insA c.9097insA	1 (7.7)
ERCC5	chr13:103524611	p.K150fs p.K917fs	c.450delA c.2751delA	5 (38.4)
MSH6	chr2:48030639	p.F56fs p.F1088fs – p.F958fs p.F786fs	c.165delC c.3261delC c.*2608delC c.2871delC c.2355delC	6 (46.1)
RAD50	chr5:131931451	p.K722fs – – p.?661fs	c.2165delA c.*1791delA c.*351delA c.1982delA	4 (30.8)
WRN	chr8:30915970	p.K5fs	c.15delA	2 (15.4)
Multidrug resistance/xenobiotic metabolism				
ABCB1	chr7:87160618	p.S893A p.S829A	c.2677T>G c.2485T>G	3 (23.0)
CYP2B6	chr19:41515263	p.K262R	c.785A>G	6 (46.1)
CYP2C9	chr10:96702047	p.R144C	c.430C>T	1 (7.7)
CYP2D6	chr22:42524994	p.P34S p.P12S	c.100C>T c.34C>T	2 (15.4)
GSTP1	chr11:67352689	– p.I105V	c.*137A>G c.313A>G	2 (15.4)
SLCO1B1	chr12:21331549	p.V174A	c.521T>C	2 (15.4)
Cell cycle control/proliferation				
CTNNB1	chr3:41266098	p.D32V p.D25V	c.95A>T c.74A>T	1 (7.75)
FGFR2	chr10:123274794	p.Y261C p.Y147C p.Y287C p.Y263C p.Y286C p.Y375C p.Y376C p.Y377C p.Y260C –	c.782A>G c.440A>G c.860A>G c.788A>G c.857A>G c.1124A>G c.1127A>G c.1130A>G c.779A>G c.*171A>G	1 (7.75)
FGFR4	chr5:176520243	p.G23R p.G388R	c.67G>A c.1162G>A	1 (7.75)
TP53	chr17:7579447	p.G254D p.G113D p.G206D p.G152D p.G86D p.G234D	c.374G>A c.338G>A c.617G>A c.455G>A c.257G>A c.701G>A	1 (7.75)
TP53*	chr17:7579407	p.A86fs p.A47fs	c.257del279 c.140del162	1 (7.75)
Signal transduction signalling				
PIK3CA	chr3:178952085	p.H1047R	c.3140A>G	1 (7.75)
PIK3CA*	chr3:178952085	p.H1047L	c.3140A>T	1 (7.75)
SOS1	chr2:39249927	p.S548R	c.1642A>C	1 (7.75)
Chromatin structure				
ASXL1	chr20:31022441	p.G641fs p.G646fs	c.1919insG c.1934insG	3 (23.0)
Liver transcription factor				
HNF1A	chr12:121432114	p.P291fs p.G226fs –	c.864delG c.677delG c.*304delG	12 (92.3)
Apoptosis				
BAX	chr19:49458970	p.E41fs p.R24fs p.E24fs	c.121insG c.69insG c.70insG	9 (69.2)
DNA methylation				
DNMT3A	chr2:25457242	– p.R693H p.R659H p.R882H	c.*498G>A c.2078G>A c.1976G>A c.2645G>A	2 (15.4)

Chromosome location, amino acid (AA) change and codon change are indicated

(na, not available)

### Association between variants, patient’s clinicopathological characteristics and overall survival

A possible association between the genetic variants and the patient’s clinicopathological characteristics was also evaluated. Due to the little size of the cohort, patients were clustered into those with variants identified only at early time points (T1 and T2) and those with variants identified only at later time points (T3 and T4). We measured a significant association between the BAX variant and portal vein invasion (*p *= 0.014) and between the HNF1A variant and liver cirrhosis (*p *= 0.032). For both genes, the significance was found only at the earlier time points. On the contrary, CYP2B showed to be significantly (p = 0.005) associated to BCLC grading only at the later time points. No further correlations were found (all *p *> 1.0) (Table 4).

**Table 4:** Association between variant detection at different time points and patient’s clinicopathological characteristics

Variable	Patients	CYP2B6 (T1–T2)	CYP2B6 (FU)	BAX (T1–T2)	BAX (FU)	HNF1A (T1–T2)	HNF1A (FU)
	n	8	8	4	6	5	12
Portal vein invasion							
Yes	3	3	1	3	1	1	3
No	10	5	7	1	5	4	9
*p*		0.231	0.51	0.014*	1	1	1
BCLC							
A + B	7	4	7	1	2	2	7
C	6	4	1	3	4	3	5
*p*		1	0.005*	0.266	0.286	0.592	1
Metastases							
Yes	4	3	1	2	3	2	3
No	9	5	7	2	3	3	9
*p*		0.608	0.217	0.53	0.266	1	0.538
Cirrhosis							
Yes	8	6	6	3	4	1	7
No	5	2	2	1	2	4	5
*p*		0.293	0.293	0.608	1	0.032*	1
Tumor size (cm)							
< 5	6	2	4	2	2	2	6
> 5	6	5	3	2	3	2	6
*p*		0.242	1	1	1	1	1

The analysis shows that there is a significant association between variants in CYP2B6, BAX and HNF1A and BCLS status (*p *= 0.005) portal vein invasion (*p *= 0.014) and absence of liver cirrhosis (*p *= 0.032), respectively. No association was found between any variant and presence of metastases or tumor size (all *p *> 1.0). Comparison was performed (for each time group separately) using the Fischer exact test

**Fig. 7:**
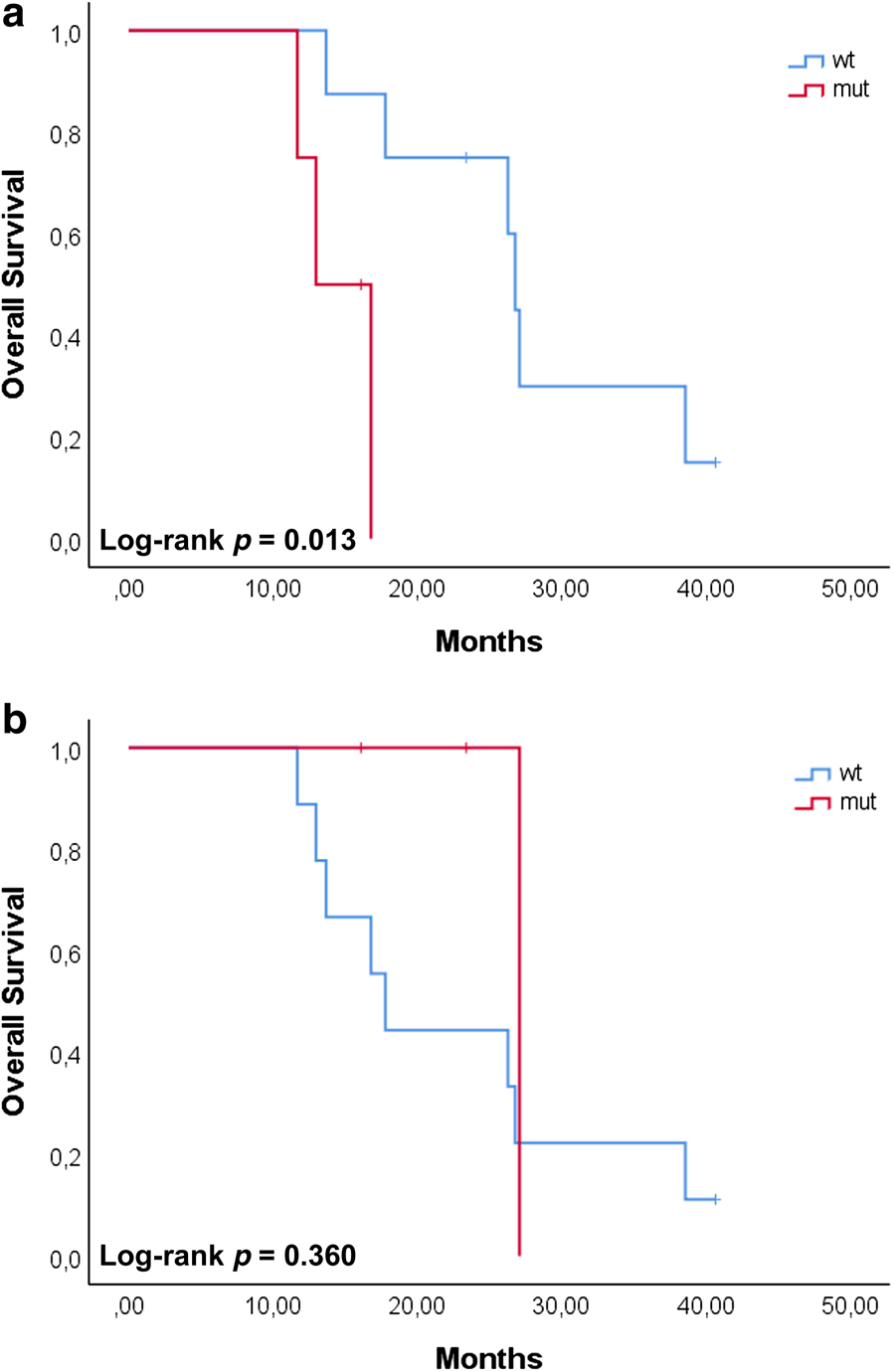
Survival plots for HCC patients carrying the CP2B6 variant. Patients were grouped according to the time points (T1–T2, **a**; T3–T4, **b**). Patients carrying the variant at T1–T2, showed a worse survival with respect to those patients carrying the variant at T3–T4. OS was analyzed using the Kaplan–Meier method and survival estimates in the different groups were compared using the log-rank test

We further evaluated the prognostic value of the genetic variants with respect to OS. Patients carrying the CYP2B6 variant in the time frame T1–T2 showed a worse OS (*p *= 0.013). On the contrary, when the same variant was detected at later time points, no significant correlation was found (*p *= 0.360) (Fig. 7). No other genetic variant showed any correlation with the clinical outcome at any of the time points considered (all *p *> 0.1).

## Discussion

HCC is a heterogeneous pathology, involving multiple somatic genomic alterations responsible for different degrees of tumor differentiation and related clinical outcomes.

TP53 and CTNNB1 are the two most frequent mutations although other driver mutations have been identified [27]. Genomic analysis, based on liver biopsy, allows the molecular subtyping of HCC and opens the door to select and adapt therapies to the individual patient’s needs. Apart from being associated to a higher risk for the patient, tissue biopsy presents nevertheless some limitations: first, the genetic heterogeneity in tumor differentiation cannot be assessed by a single site sampling; second, the histopathological assessment of vascular invasion requires large biopsies; third, being highly invasive, biopsy is usually performed only once during the whole therapy. Liquid biopsy is independent of these limitations and shows predictive and prognostic value in several types of cancer including HCC [12, 13, 28]. cfDNA quantitative changes have been mainly linked to tumor’s burden and therapeutic efficacy [26], while ctDNA qualitative changes can detect genetic variations in the tumor as a whole [16, 29].

In this exploratory study we investigated the prognostic value of cfDNA/ctDNA isolated from advanced HCC patients over multiple time points in the context of the SORAMIC trial. We observed that the amount of cfDNA measured immediately after micro-intervention significantly correlated with the presence of distant metastases, supporting also in this clinical context a diagnostic value of cfDNA to unravel secondary lesions, as showed in other tumor types [25, 30, 31]. In addition, patients with post-operative high levels of cfDNA had a shorter OS. Interestingly, this holds true only at the later time points, after several weeks of sorafenib treatment, suggesting that also in advanced HCC cfDNA can support in monitoring therapy response. This finding is relevant when considering the use of other biomarkers such as AFP [32, 33] whose prognostic value is still under debate [34, 35]. Among the patients enrolled in this study, only two patients had, over the considered time points, AFP concentrations higher than the assigned cut-off value of 400 ng/ml. On the contrary, quantitative analysis of post-operative cfDNA was always feasible and showed a trend with the clinical history of the patients, supporting the use of cfDNA to monitor therapy response also in advanced HCC.

NGS analysis showed that all 13 patients presented SNV and/or InDel variants in a total of 28 genes, with 6 as the median number of variants per patient. We never found two patients with an identical profile, supporting the concept that HCC is a highly heterogeneous disease. Interestingly, only in few cases we detected the hotspot variants normally associated to HCC as those in TP53 or CTNNB1. We cannot exclude that this result is due to the low number of patients included in the study, therefore a validation study with a larger cohort will be necessary. However, these driver mutations are usually associated to specific etiological characteristics, not present in our patients’ group, such as HBV infection, dietary exposure to aflatoxin B1, or are per se very rare events [36]. The absence of these variants could be therefore associated to the clinical characteristics of the patients. In addition, in a multimodal meta-analysis run over 1494 HCC samples, it has been shown that other driver mutations, less frequent nevertheless still clinically significant, can be identified [37]. From the 28 variants, we identified in particular 3 genes displaying high MAFs. CYP2B6 (mutated in 46.1% of the patients at least at one time point) belongs to the Cytochrome P450 class of enzymes which are important catalysts among all metabolizing enzymes. The isoform CYP2B6, expressed mainly in the liver, is highly polymorphic [38] (with the haplotype CYP2B6*6 found in the Caucasian population in up to 25% of the cases) and shows clinical relevance [38, 39]. However, there are contradictory results on the effect of the haplotype *6 (h*6). On one side it has been reported that patients carrying h*6 have a reduced amount of mRNA and protein [40], with an inferior response to specific treatments. On the other side, studies demonstrated that h*6 increase the enzymatic activity of CYP2B6 [41]. In our study, we found that 46% of the patients presented a CYP2B6 variant corresponding to h*6 and that the detection of this variant at earlier time points was significantly associated with a worse prognosis and a shorter OS. Since variants in enzymes involved in the metabolism can affect the response to specific drugs [42] and CYP450 and its haplotypes are involved in the metabolism of several drugs [39, 43], we can hypothesize that those patients presenting the variant h*6 responded worse to the therapy with sorafenib and therefore had a short OS.

BAX (mutated in 69.2% of the patients at least at one time point) belongs to the Bcl-2 gene family of pro-apoptotic proteins [44]. Inactivation of BAX might be a mechanism to escape cell death, providing a survival advantage to the tumor. In colorectal cancer this particular frameshift mutation is indeed conferring an advantage to tumor progression, with p53-independent induction of apoptosis [45]. Sorafenib is a multikinase inhibitor with a strong anti-proliferative and anti-angiogenic effect on different cell types [46], displaying also a pro-apoptotic effect [47]. BAX might play a role in cellular death induced by sorafenib, and the presence of this frameshift variant might predict a low therapy efficacy due to the inactivation of the pathway inducing apoptosis. Although we did not find any significant correlation between the presence of variants of BAX and OS, we unraveled a significant correlation with portal vein invasion. Tumor cells able to overcome apoptosis display higher invasive characteristics. The fact that 3 patients carrying this mutation are positive for portal vein infiltration might support the role of BAX in tumor invasion and the possible inefficacy of sorafenib in these patients. However at the moment this is just a hypothesis and further analysis will be necessary to confirm it.

Finally, HNF1A showed the highest MAF, reaching 92.3% over the whole cohort. HNF1A codes for a transcription factor (HNF1α) which regulates liver differentiation and development [48]. This transcription factor functions as a tumor suppressor [49] and its inactivation might play an important role in tumor development [50]. A recent work of Takashima et al., demonstrated that the ectopic expression of HNF1α combined to other two transcription factors (HNF4a and FOXA3) successfully inhibited proliferation in a cellular model system [51]. If confirmed in other model system, HNF1A could be a novel target for combined therapy, especially when drug resistance is developed.

Moreover, while HNF1A inactivation is known to be frequent in hepatocellular adenoma, somatic mutations in this gene are relatively a rare event in HCC and very often bound to specific etiological characteristics such as absence of cirrhosis and viral infection [52]. In our cohort, none of the patients but one presented with viral infection and the presence of the variant showed a significant negative association with cirrhosis. Although TP53 and CTBNN1 are the two most frequently mutated genes in HCC, there are other low frequency driver genes, such as HNF1A, which nevertheless are significantly associated to patient’ survival. We can hypothesize that in the patient cohort selected for this study, HNF1A was a driver mutation. If confirmed in a larger number of patients, HNF1A could represent a novel target for personalized therapy.

A remarkable result we observed was the dynamic change of the mutation status over time. With respect to each gene taken individually, in the time frame between T1 and T4 we identified 6 categories of patients, showing: always the wildtype gene (WT); always the mutated gene (M); a single shift WT → M; a single shift M → WT; a double shift WT → M → WT; a double shift M → WT → M. With the small number of patients included in the study, for the moment we can only speculate that these changes are mirroring clonal heterogeneity found in the primary tumors [53, 54], evidenced because the analysis was done by liquid biopsy. It is reasonable to speculate that the shift WT → M might take place because the mutated clones are more resistant to therapy; therefore post-treatment samples are enriched with ctDNA carrying the mutation. These clones possibly develop spontaneously and the mutation they carry confers a selective advantage to cells. On the contrary, the shift M → WT might be observed because the mutated clones are more sensitive to therapy, which possibly could slow down their proliferation, while WT clones divide and expand faster. In the latter case we cannot exclude that ctDNA carrying the mutation is still present, however in this case the corresponding MAF is under the NGS detection threshold. Dynamic changes in clonal expansion during therapy are a known event [55], also strongly suggested by our data. Due to the limited number of samples, we cannot provide here anything more than a hypothesis. However it will be surely interesting to pursue in this direction, enlarging the number of cases and validating these preliminary results.

## Conclusions

A validation study with a larger cohort and the inclusion of appropriate control groups will be necessary to confirm these preliminary results and to support their clinical value. Also a more detailed comparison between patients receiving ablation or radio-embolization should be considered to understand if different procedures possible with the micro-interventional therapy might induce the accumulation of alternative genetic mutations. However, from the results obtained from this exploratory study we can conclude that also in advanced HCC after micro-intervention cfDNA quantification is feasible, can support standard imaging analysis for the early detection of metastatic lesions and is showing a prognostic value with respect to OS. In addition, interrogating ctDNA for genetic variants can reduce understaging of clonal selection as a consequence of systemic therapy, in the potential to improve longitudinal monitoring of therapy efficacy. HCC is a highly heterogeneous tumor type; it would be therefore an advantage to tailor therapy according to the intrinsic genetic characteristics. None of the patients included in this study showed an identical genomic profile, supporting the concept that also in advanced HCC personalized therapy is necessary to improve clinical outcome. A rapid adaption of therapy in concomitance to variant appearance and therefore a more efficient treatment might be particularly important for patients already in an advanced stage of the disease and therefore with a relative short overall survival expectancy.

## Supplementary information

**Additional file 1: Figure S1.** Size distribution of extracted cfDNA after capillary electrophoresis (CE). DNA extracted from all the plasma samples were fractionated by CE and analyzed. cfDNA displays a size of approximately 150 base pairs (bp), corresponding to one nucleosome, and multiple of this size, while gDNA has a higher molecular weight of several kilobases (kb). Samples displaying a unique peak around 150 bp were further analyzed, while samples showing a peak several kb were no further processed. Panels A and B report examples of cfDNA and gDNA as determined by CE, respectively (RFU, relative fluorescence unit; LM, lower marker; UM, upper marker).

**Additional file 2: Table S1.** Depth of coverage. An average coverage of at least 1000 times or more was reached for all the samples (average 1743.63, range 1182.17–2870.64), except one (sample F4) which showed a lower depth (840.41).

**Additional file 3: Table S2.** List of variants as detected in genomic DNA extracted from buffy coats for each patient. Chromosome location, gene name and mutation frequency are indicated. The analysis was done for all the patients except for patient E, whose corresponding cellular sample was not available. For this patient we set arbitrarily a mutation frequency threshold corresponding to the average of the other samples. Genes presenting a mutation frequency of 50 (± 5) and 100 (± 5) % were considered carrying a germline mutation and therefore were excluded from the following analysis.

## Data Availability

The data supporting the conclusions of this article are included within the article.

## Abbreviations

HCC: hepatocellular cancer
cfDNA: circulating free DNA
ctDNA: circulating tumor DNA
NGS: next generation sequencing
AFP: alpha-fetoprotein
SNV: single nucleotide variants
InDel: insertions and deletions
