# Supplementary figures and images for: Circulating DNA as prognostic biomarker in patients with advanced hepatocellular carcinoma: a translational exploratory study from the SORAMIC trial
Source: J Transl Med. 2019 Oct 1;17:328. doi: 10.1186/s12967-019-2079-9 (PMC6771167; doi:10.1186/s12967-019-2079-9)

A

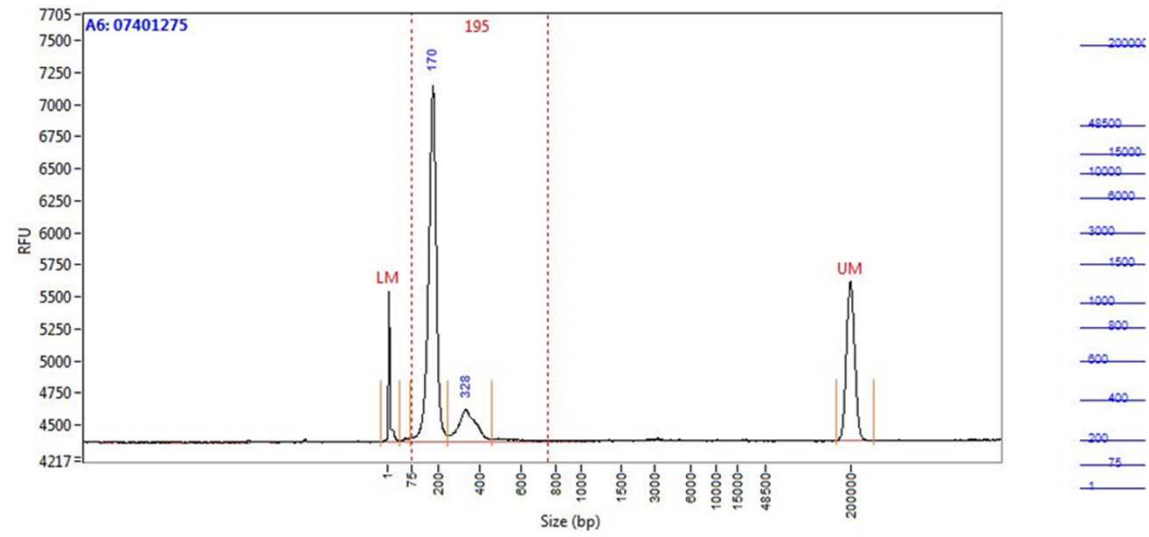

B

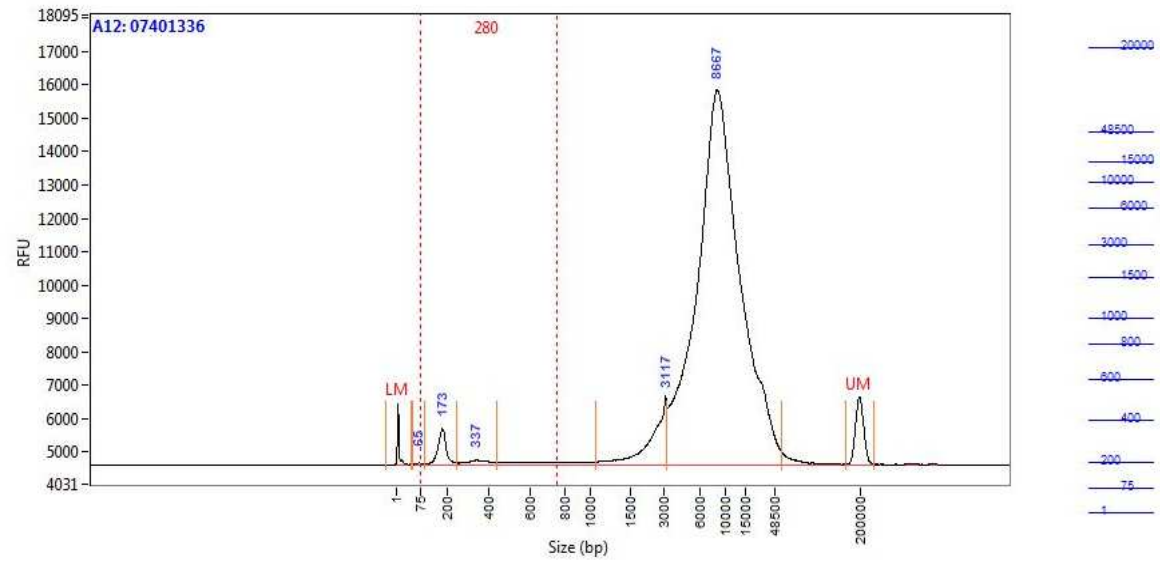

Supplement: Supplementary file 1 — Additional file 1: Figure S1. Size distribution of extracted cfDNA after capillary electrophoresis (CE). DNA extracted from all the plasma samples were fractionated by CE and analyzed. cfDNA displays a size of approximately 150 base pairs (bp), corresponding to one nucleosome, and multiple of this size, while gDNA has a higher molecular weight of several kilobases (kb). Samples displaying a unique peak around 150 bp were further analyzed, while samples showing a peak several kb were no further processed. Panels A and B report examples of cfDNA and gDNA as determined by CE, respectively (RFU, relative fluorescence unit; LM, lower marker; UM, upper marker). [file 12967_2019_2079_MOESM1_ESM.pdf]
