# Supplementary material for: Circulating DNA as prognostic biomarker in patients with advanced hepatocellular carcinoma: a translational exploratory study from the SORAMIC trial
Source: J Transl Med. 2019 Oct 1;17:328. doi: 10.1186/s12967-019-2079-9 (PMC6771167; doi:10.1186/s12967-019-2079-9)
Supplement: Supplementary file 2 — Additional file 2: Table S1. Depth of coverage. An average coverage of at least 1000 times or more was reached for all the samples (average 1743.63, range 1182.17–2870.64), except one (sample F4) which showed a lower depth (840.41). [file 12967_2019_2079_MOESM2_ESM.docx]

Table S1

| **Target coverage** | | | | |
| --- | --- | --- | --- | --- |
| **Sample** | **Average (X)** |  | **Sample** | **Average (X)** |
| A1 | na |  | H1 | na |
| A2 | 2003,74 |  | H2 | 1569,03 |
| A3 | 1425,20 |  | H3 | 2001,63 |
| A4 | 1432,66 |  | H4 | 2870,64 |
| B1 | 1415,15 |  | I1 | 1897,08 |
| B2 | 1182,17 |  | I2 | 1876,22 |
| B3 | na |  | I3 | 1816,58 |
| B4 | 1632,34 |  | I4 | 1692,61 |
| C1 | 2607,23 |  | J1 | 1503,98 |
| C2 | 2651,16 |  | J2 | na |
| C3 | 1643,36 |  | J3 | 1823,92 |
| C4 | 2224,80 |  | J4 | 1735,20 |
| D1 | na |  | K1 | na |
| D2 | 1826,46 |  | K2 | 1460,72 |
| D3 | 2133,22 |  | K3 | 1428,69 |
| D4 | 1392,37 |  | K4 | 2101,73 |
| E1 | 1561,46 |  | L1 | 1587,94 |
| E2 | 1574,69 |  | L2 | 1910,78 |
| E3 | 1447,09 |  | L3 | 1803,57 |
| E4 | 1429,32 |  | L4 | 1997,29 |
| F1 | 1586,12 |  | M1 | na |
| F2 | 1640,46 |  | M2 | na |
| F3 | 1302,98 |  | M3 | na |
| F4 | 840,41 |  | M4 | 1782,40 |
| G1 | 1645,48 |  |  |  |
| G2 | 1305,47 |  |  |  |
| G3 | 1655,56 |  |  |  |
| G4 | 1654,15 |  |  |  |
