# Supplementary material for: Circulating DNA as prognostic biomarker in patients with advanced hepatocellular carcinoma: a translational exploratory study from the SORAMIC trial
Source: J Transl Med. 2019 Oct 1;17:328. doi: 10.1186/s12967-019-2079-9 (PMC6771167; doi:10.1186/s12967-019-2079-9)
Supplement: Supplementary file 3 — Additional file 3: Table S2. List of variants as detected in genomic DNA extracted from buffy coats for each patient. Chromosome location, gene name and mutation frequency are indicated. The analysis was done for all the patients except for patient E, whose corresponding cellular sample was not available. For this patient we set arbitrarily a mutation frequency threshold corresponding to the average of the other samples. Genes presenting a mutation frequency of 50 (± 5) and 100 (± 5) % were considered carrying a germline mutation and therefore were excluded from the following analysis. [file 12967_2019_2079_MOESM3_ESM.docx]

Table S2

| **Chromosome**  **location** | **Gene** | **Patients** | | | | | | | | | | | | |
| --- | --- | --- | --- | --- | --- | --- | --- | --- | --- | --- | --- | --- | --- | --- |
|  |  | **A** | **B** | **C** | **D** | **E** | **F** | **G** | **H** | **I** | **J** | **K** | **L** | **M** |
| chr10:96541616 | AL583836.1 |  |  |  |  |  |  |  | 48,4 |  | 46,3 |  |  |  |
| chr20:31022441 | ASXL1 |  |  |  |  |  |  |  | 1,8 | 2 |  |  |  |  |
| chr7:87160618 | ABCB1 | 100,00 | 100,00 |  | 45,7 |  | 99,8 | 100 |  | 99,6 | 53,5 |  | 49,5 | 54,4 |
| chr19:49458970 | BAX | 1,80 |  |  |  |  | 1,6 |  |  |  |  |  |  |  |
| chr15:93545433 | CHD2 |  |  |  | 1,5 |  | 2,6 |  |  |  |  |  |  |  |
| chr19:41512841 | CYP2B6 |  | 52,20 |  |  |  | 46,7 |  |  |  | 48,9 |  | 44,5 | 50,5 |
| chr19:41515263 | CYP2B6 |  | 18,50 |  |  |  | 38,2 | 12,4 |  |  | 42,3 |  | 34,5 | 33 |
| chr10:96702047 | CYP2C9 | 49,40 | 40,40 |  |  |  |  | 45,1 |  |  |  |  |  |  |
| chr22:42524947 | CYP2D6 |  |  | 46,6 |  |  |  | 62 | 50 |  | 42,7 | 48,2 |  |  |
| chr22:42526694 | CYP2D6 |  |  | 71,1 |  |  |  | 66,7 |  |  | 73,3 |  |  |  |
| chr1:98165091 | DPYD |  |  |  |  |  | 52 |  | 45 |  |  |  | 51,2 | 49,8 |
| chr5:176520243 | FGFR4 | 46,10 |  | 54,9 |  |  | 48,4 |  | 52,9 |  | 100 | 54,3 |  |  |
| chr11:67352689 | GSTP1 | 47,50 | 44,90 | 99,7 |  |  |  | 50 | 47,3 |  | 50,3 |  | 99,6 | 46,8 |
| chr12:121432117 | HNF1A |  |  |  |  |  |  |  | 1,4 |  | 1,4 |  |  |  |
| chr1:43804305 | MPL |  |  |  |  |  |  |  |  |  |  | 40 |  |  |
| chr12:21331549 | SLCO1B1 |  |  |  |  |  |  |  |  |  |  |  | 51,5 | 49,8 |
| chr7:141672604 | TAS2R38 | 99,70 | 99,80 | 100 | 50,7 |  | 47,5 | 54,4 | 100 | 49,3 | 52,6 | 46,7 |  | 99,8 |
| chr7:141673345 | TAS2R38 | 99,30 | 99,80 | 100 | 55,2 |  | 49,6 |  | 99,8 | 48,5 | 50,4 | 48,9 |  | 100 |
| chr17:7579472 | TP53 | 46,60 | 51,60 | 98,9 | 100 |  | 100 | 99,6 | 49,2 | 45,3 | 100 | 99,1 | 100 |  |
| chr3:14187449 | XPC | 42,70 | 100,00 | 100 | 99,4 |  | 100 | 47,4 | 100 | 42,3 | 100 | 100 | 46,8 | 51,6 |
| chr12:21331549 | SLCO1B1 |  |  |  |  |  |  |  | 48,4 |  | 46,3 |  |  |  |
| chr7:141672604 | TAS2R38 |  |  |  |  |  |  |  | 1,8 | 2 |  |  |  |  |
| chr7:141673345 | TAS2R38 | 100,00 | 100,00 |  | 45,7 |  | 99,8 | 100 |  | 99,6 | 53,5 |  | 49,5 | 54,4 |
